# Exploring Innovative Strategies to Enhance Electronic Health Record Interoperability in U.S. Healthcare Settings

**DOI:** 10.3390/healthcare14101285

**Published:** 2026-05-09

**Authors:** Craig McPherson, Reece Davis, Manasa Battu, Bruce Lazar

**Affiliations:** School of Health Sciences, Southern Illinois University System, Carbondale, IL 62901, USA; reece.davis@siu.edu (R.D.); manasa.battu@siu.edu (M.B.)

**Keywords:** health information interoperability, artificial intelligence, electronic health records, health information exchange, continuity of patient care

## Abstract

**Objectives**: Improving the interoperability of electronic health records is critical for efficient, cost-effective delivery of quality services, enhanced care coordination, and improved treatment outcomes within the United States healthcare system. Healthcare leaders and administrators often experience EHR interoperability issues, restricting communication between health systems and impacting electronic health data utilization. **Methods**: This systematic literature review explored innovative strategies to improve electronic health record interoperability between health information systems to enhance data exchange efficiency, accuracy, and security in U.S. healthcare settings. A search transpired using the Public Medline, Institute of Electrical and Electronics Engineers Xplore Digital Library, and Cumulative Index to Nursing and Allied Health Literature following the Preferred Reporting Items for Systematic Reviews and Meta-Analyses guidelines. **Results**: Data from 24 relevant articles were analyzed using screening criteria revolving around the research question. Five themes emerged during data analysis. The themes included the utilization of blockchain-based EHR systems (67%), the drive of the Cures Act to achieve interoperability (17%), the advent of artificial intelligence and how it can be used (33%), how the Internet of Things drives the industry to strategically enhance the system (33%), and how the value of interoperability drives outcomes (79%). **Conclusions**: Findings indicate strategies from a technical perspective and from policy initiatives can improve communication between health information systems. Findings suggest that by strategically leveraging available resources and implementing innovative strategies, healthcare leaders can achieve comprehensive EHR interoperability long term.

## 1. Introduction

In today’s evolving model of healthcare delivery, a complete exchange of electronic health records (EHRs) stands at a critical juncture for improving patient care outcomes, operational efficiency, and healthcare quality. However, despite significant advancements in health information technology, interoperability between the vast number of health information systems remains the largest obstacle within the United States healthcare framework [1]. Interoperability is the capability of various information systems, devices, or applications to link, share, and trade data seamlessly in a synchronized manner, which is fundamental for facilitating the seamless flow of patient information across various healthcare entities [2,3]. According to Kumari and Chander, improving the interoperability of electronic health records within the U.S. healthcare system is important for efficient, cost-effective delivery of quality services, enhanced care coordination, and better treatment outcomes [4]. Healthcare leaders and administrators face challenges with the interoperability of EHRs that hinder communication between various health information systems and negatively affect the utilization of electronic health data for decision-making, security, effective care coordination, and treatment outcomes [5,6].

The research problem is that healthcare leaders need innovative strategies to improve the interoperability of electronic health record communication between health information systems to enhance data exchange efficiency, accuracy, and security in U.S. healthcare settings. Several authors have stated that the introduction of newer technologies to the healthcare system leads to emerging issues, including integrating older systems, addressing privacy and data security concerns, managing fragmented systems, high implementation costs, and coping with limited IT resources, all of which combined could influence the interoperability of EHRs [7]. However, despite these challenges, some researchers argue that the benefits of innovative technology and the goal of interoperability in EHRs outweigh any potential barriers for the healthcare system and the patients it serves. Therefore, the systematic literature review aims to explore innovative strategies to improve the interoperability of electronic health record communication between health information systems to enhance data exchange efficiency, accuracy, and security in U.S. healthcare settings.

## 2. Methods

After identifying and selecting the research topic, a well-crafted selection of articles relevant to the research question occurred. The research question was, what innovative strategies can healthcare leaders implement to improve the interoperability of electronic health record communication between health information systems to enhance data exchange efficiency, accuracy, and security in U.S. healthcare settings? A search of the literature transpired using Public Medline (PubMed), Institute of Electrical and Electronics Engineers Xplore Digital Library (IEEE Xplore), and Cumulative Index to Nursing and Allied Health Literature (CINAHL Plus) in accordance with the Preferred Reporting Items for Systematic Reviews and Meta-Analyses (PRISMA) guidelines [8,9]. The Method phase included:

1. Searching for relevant studies.
2. Screening for inclusion and exclusion criteria.
3. Extracting data based on the screening criteria.
4. Synthesizing the data to identify key themes.
5. Reporting and disseminating the findings [9].

Searching the academic databases utilizing keywords, EHR, electronic health records, EMR, electronic medical records, interoperability, interoperability in healthcare, improvement, development, and advancement gave a specific framework for selecting ideal articles to help answer the research question. For consideration in the systematic literature review, the following conditions were required:

- Publication between 2020 and 2024.
- English language.
- Peer-reviewed journal articles.
- Full-text availability.
- Related to the research question centered on strategies to improve interoperability of electronic health record communication between health information systems.

**Figure 1:**
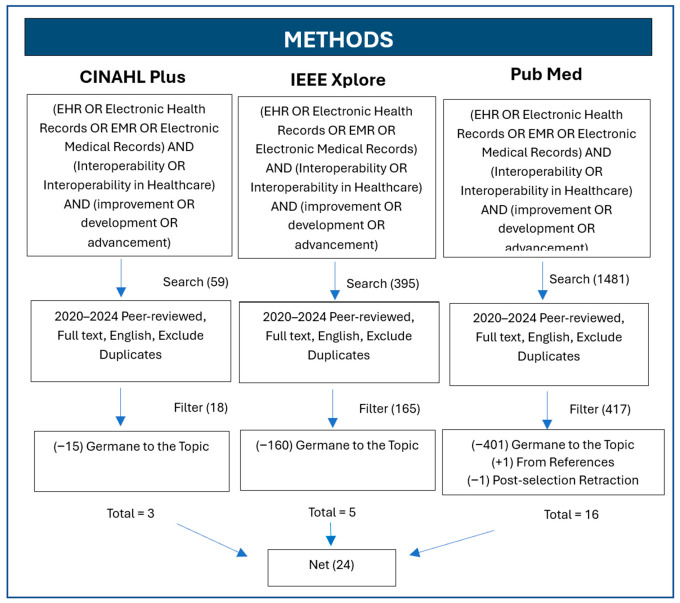
Article Screening and Selection Workflow Process. Note: One study was retracted by the publisher following the identification and screening phases and was removed from the figure.

**Figure 2:**
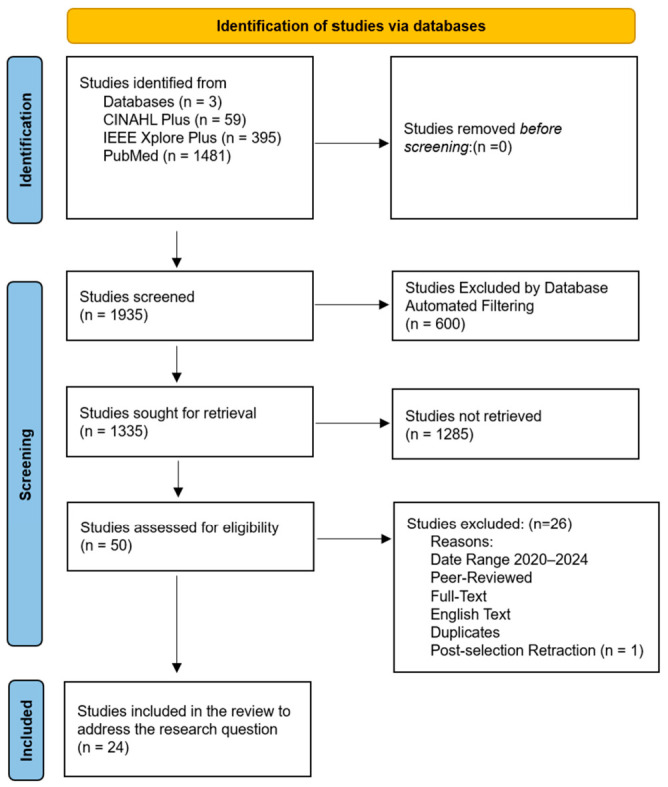
PRISMA 2020 flow diagram for systematic review, including searches of databases. Note: A study was retracted by the publisher after the identification and screening phases were completed, leading to its removal from the figure.

Boolean research searches were used for all databases (see Figure 1): (EHR OR Electronic Health Records OR EMR OR Electronic Medical Records) AND (Interoperability OR Interoperability in Healthcare) AND (improvement OR development OR advancement). Upon applying the inclusion criteria. PubMed provided 1481 articles, IEEE Xplore provided 395 articles, and CINAHL Plus yielded 59 articles, totaling 1935 records. All studies went through a screening process to make sure they were relevant to the research topic and could address the research question. One thousand, two hundred and eighty-five studies were excluded during the title and abstract screening for not meeting the eligibility criteria established and presented in Section 2. The 50 full-text articles remaining were reviewed carefully, resulting in the exclusion of additional articles based on the lack of articles specifically either germane to the topic or that could answer the research question. This systematic screening process produced twenty-five articles that were selected for inclusion in the study as shown in Figure 1 and Figure 2 for additional details concerning exclusion and the screening phases. Additionally, the details from Figure 1 and the methods were synthesized into the PRISMA 2020 flow diagram (Figure 2) for standardization purposes, offering a high-level overview of the study selection process, including identification, screening, and inclusion. Additionally, one article was discovered as retracted by the publisher before publication and was excluded from the study, bringing the total number of articles included in the study to 24.

This systematic literature review focuses on health information technology and electronic health record interoperability strategies rather than clinical interventions or patient-level outcomes, and it does not include a meta-analysis. All methods, including the search strategy, inclusion and exclusion criteria, screening process, data and thematic synthesis, were predefined and documented before data collection in accordance with the Preferred Reporting Items for Systematic Reviews and Meta-Analyses (PRISMA) guidelines, ensuring transparency and reproducibility.

Although the protocol was not registered in a public registry, all methodological decisions were established and documented in advance.

A Kappa calculation was conducted to enhance the reliability of article usage and the study’s findings. The screening was conducted in two stages: an initial independent blind review followed by a reconciliation session to resolve discrepancies. Two independent reviewers were used to review and evaluate each article for its relevance to the topic and ability to help answer the research question. The Kappa coefficient was calculated using the following guidelines: (a) 0 = no agreement, (b) 0.01–0.20 = slight agreement, (c) 0.21–0.40 = fair agreement, (d) 0.41–0.60 = moderate agreement, (e) 0.61–0.80 = substantial agreement, and (f) 0.81–1.00 = almost perfect agreement [10]. During the initial independent screening, the reviewers achieved a substantial agreement (Kappa = 0.88). Following the independent evaluation, a reconciliation meeting occurred to discuss and resolve any initial discrepancies. However, there were no discrepancies between the independent review, with a final observed agreement of 100% meeting the inclusion criteria and timeframe required as shown in Table 1. The final Kappa value of 1.0 was influenced by the objective nature of the inclusion and exclusion criteria established in the study (See Section 2) and was based on the 25 articles evaluated during the screening phase. One study was later removed due to a retraction from the publisher before publication, which resulted in 24 studies.

## 3. Results

The primary research question that guided the study was, what innovative strategies can be implemented by healthcare leaders to improve the interoperability of electronic health record communication between health information systems to enhance data exchange efficiency, accuracy, and security in U.S. healthcare settings? Searching three academic electronic databases: Public Medline (PubMed), Institute of Electrical and Electronics Engineers Xplore Digital Library (IEEE Xplore) and Cumulative Index to Nursing and Allied Health Literature (CINAHL Plus), yielded numerous studies. The review adhered to an established literature search strategy, selection procedure, and data analysis technique, as noted in the Preferred Reporting Items for Systematic Reviews and Meta-Analyses [8,9]. Based on the data from 1935 studies chosen, 25 articles were germane to the topic and relevant to the research question. However, one study was excluded due to a publisher-issued retraction before publication. Twenty-four studies were included in the analysis. A final choice was made by systematically examining the summary findings extracted from each article (see Figure 1 and Figure 2). Table 2 provides an overview of these twenty-four selected studies’ titles and detailed perspectives on the findings.

The data obtained from the 24 articles, which classified the occurrence frequency of vital occurrences such as *blockchain-based electronic health records (EHR) systems*, *how the Cures Act impacts strategies of interoperability implementation*, *use of artificial intelligence*, *the advancement of the Internet of things (IoT)*, *and the value of interoperability overall within the healthcare world and patients’ health information*, led to the development of five main themes. Each theme directly relates to the research question. The following themes included (a) Blockchain-Based EHR Systems; (b) the Cures Act; (c) Artificial Intelligence; (d) the Internet of Things; and (e) the value of Interoperability.

From the research findings, 64% of the articles provided documented information regarding Blockchain Based EHR systems (Occurrences: 1, 3, 4,5, 6, 7, 9, 10, 11, 13, 14, 15, 20, 21, 23). 16% of the articles addressed how the Cures Act impacts interoperability implementation (Occurrences: 9, 16, 17, 18). 32% of the articles outline how artificial intelligence can be used in different ways to achieve interoperability and quality care (Occurrences: 2, 4, 11, 13, 19, 20, 22, 24). The data also showed that 32% of the articles addressed the newest aspect of healthcare by outlining how the Internet of Things must be taken into consideration as part of the overall strategy (Occurrences: 1, 3, 11, 13, 14, 20, 22, 24). Finally, the strongest theme throughout the twenty-five articles was the focus on the value of interoperability (Occurrences: 2, 4, 5, 6, 7, 8, 9, 10, 12, 13, 15, 16, 17, 18, 19, 20, 22, 23, 24).

## 4. Discussion

This systematic literature review explored innovative strategies to improve the interoperability of electronic health record communication between health information systems to enhance data exchange efficiency, accuracy, and security in U.S. healthcare settings. Twenty-five peer-reviewed contemporary articles that were published between 2020 through 2024 were considered in the study, which allowed for a current analysis of blockchain-based EHRs, the involvement of Artificial Intelligence, the Internet of Things entering the market, the government’s Cures Act and the overall connectedness of interoperability communication between systems. Frequency-based analysis counts were used to identify key areas of industry focus, followed by a thematic synthesis to evaluate readiness for practical application and adoption. The data results shown in Table 3 display the main themes that emerged from the literature analysis. Five main themes emerged from the literature analysis that included blockchain-based EHR systems (Occurrences: 1, 2, 3, 4, 5, 6, 7, 9, 10, 11, 13, 14, 15, 20, 21, 23), the Cures Act (Occurrences: 9, 16, 17, 18), artificial intelligence (Occurrences: 2, 4, 11, 13, 19, 20, 22, 24), Internet of Things (Occurrences: 1, 3, 11, 13, 14, 20, 22, 24) and interoperability/connectedness (Occurrences: 2, 4, 5, 6, 7, 8, 9, 10, 12, 13, 15, 16, 17, 18, 19, 20, 21, 23, 24).

Theme one, as represented in 67% of the articles, generated data on blockchain-based EHR systems. The higher frequency-based count indicates significant concurrence in the available literature suggesting that the use of blockchain technology in electronic health records and patient-centered interoperability can drive healthcare innovation significantly, forming a formidable strategy [21]. Researchers’ findings in one study concluded that the system’s use of blockchain technology enhances data management, sharing, and security more effectively than conventional EHR systems [12]. A positive aspect found in the results was that regardless of where the healthcare practitioner was physically located, they were still able to access their patient’s records and medical information [12]. While blockchain-based EHR systems are important, the Cures Act which is driven by the U.S. government helps drive these innovations.

Theme two, as demonstrated by 17% of the studies, focused on the Cures Act, which aims to create a competitive ecosystem of third-party apps that can be written once and connected to standardized health system data anywhere. Today the Cures Act could be used as a strategic basis because many EHR products are based on pre-internet software but can seize the opportunity to layer on a modern infrastructure and use it as a pathway forward. Even though the percentage of studies reported was lower, the Cures Act is an actionable and standardized implementable strategy because it provides established guidelines for immediate adoption into practice. A significant concern highlighted by the results is the possibility of vendors limiting functionality and the availability of data by repeating behaviors experienced in the past [28]. While government acts and laws help drive the progression of interoperability, a lesser-known advancement in technology is the utilization of artificial intelligence.

Artificial intelligence emerged as the third theme, noted in 33% of the articles, as a possible strategic perspective that healthcare leaders can use to improve the interoperability of electronic health record communication between health information systems. The frequency-based count indicates that AI is observed as a helpful and valuable tool for improving interoperability, but it cannot independently be the solution to significant interoperability issues. Artificial Intelligence and advanced analytics can provide valuable insights into the vast patient data stored in a blockchain-based cloud EHR system [12]. Artificial intelligence and digital data have become crucial in the era of big data for everyday functioning and healthcare services. Artificial intelligence could not only be beneficial for interoperability but also integrated with the Internet of Things, ensuring its incorporation into the EHR [21].

In the realm of healthcare, leveraging the fourth theme mentioned in 33% of the studies, the Internet of Things (IoT) represents a pivotal strategic approach aimed at enhancing the interoperability of Electronic Health Records (EHR), transforming how patient data is accessed, shared, and utilized across healthcare systems [11]. The integration of Blockchain Technology, a scalable IoT framework, and a pioneering mining mechanism has significantly accelerated the development of Electronic Health Records Management [11]. The integration of Blockchain and IoT identified in the analysis suggests a more decentralized shift for IoT data management, supporting more precise predictive models and decision support systems through improved traceability, while emphasizing the importance of communication between health information systems [13]. Having access to this latest technology could lead to improved communication, resulting in strong interconnectedness and interoperability.

The last theme was consistently mentioned across studies, occurring in 79% of the articles, which suggests a consensus that the value of interoperability is a strategic priority for the field. By addressing the challenges of EHR interoperability and embracing the potential of advanced implementations, healthcare leaders can significantly improve the quality and efficiency of care delivery. The concurrence noted above suggests this strategy emphasizes the importance of a unified EHR system, which leads to improved quality and efficiency of care [4]. While many are encouraged by the prospects of open communication between healthcare systems, there is a long way to go, and those in power must understand the values gained from such a structure [22].

Overall, themes one through five suggest that improving interoperability in EHR communication calls for an integrated strategy that incorporates the adoption of blockchain-based EHR systems to improve data security, alignment with the Cures Act to support regulatory compliance and data sharing, and the inclusion of artificial intelligence and IoT technologies to enable advanced analytics, real-time data exchange, and improved clinical decision-making. All together, these components might support improvements in data exchange efficiency, accuracy, and security within U.S. healthcare systems.

Several limitations existed within this systematic literature review. The review applied an extensive search within PubMed (Public Medline) and the Cumulative Index to Nursing and Allied Health Literature (CINAHL Plus) academic databases to locate peer-reviewed journal articles published between 2020 and 2024. The scope of the review was restricted to English-only articles. Additionally, while the search process involved using Boolean searches, relevant keywords, and filters, additional articles could have been discovered in the academic databases by employing alternative terminology or filters. Finally, the subjective nature of the human aspect of reviewing articles allows for different interpretations of the same findings, which was minimized through a reconciliation process.

Another limitation of the review was that an appraisal tool, such as the JBI Critical Appraisal Tool, did not occur to score each of the included studies for risk of bias. The study focused on thematic synthesis instead of a meta-analysis of clinical outcomes, and the main focus was on the identification of interoperability strategies and solutions. The inclusion criteria were limited to peer-reviewed literature from credible databases within the last five years to establish scholarly stringency. However, without a formal risk of bias assessment, the studies were synthesized based on thematic relevance (not methodological weighting) and should be understood when evaluating the identified strategies.

To mitigate these limitations, adherence to PRISMA-based systematic review guidelines [9] was rigorous, aiming to reduce the review’s limitations. A total of 1540 sources were collected, and several filters were applied during the searches in PubMed and CINAHL databases until no additional relevant information remained to advance the research topic. Each article underwent meticulous review, resulting in twenty-five remaining articles deemed pertinent to the topic and relevant to the research question. Two independent reviewers conducted a Kappa calculation to improve the reliability of chosen articles and the study’s results. The Kappa value calculated from two independent reviewers for selected articles in the study was 1.0 [34,35].

Acknowledging the presence of limitations in the study, the findings indicate the existence of innovative strategies that healthcare leaders can implement based on the five themes that emerged from the literature. These strategies aimed to improve the interoperability of electronic health record communication between health information systems to enhance data exchange efficiency, accuracy, and security in U.S. healthcare settings.

The review’s findings can serve as a foundation to build upon for future researchers incorporating a more comprehensive research method or design that integrates qualitative interviews and quantitative surveys to provide additional insights into the possible strategies healthcare leaders may implement to improve the interoperability of electronic health record communication between health information systems to enhance data exchange efficacy, accuracy, and security in the U.S. healthcare system. Also, policymakers could use these results as a stepping-stone to develop policies related to a nationwide plan to improve interoperability of electronic health record communication between health information systems in the United States. Findings indicated that healthcare leaders are working to implement innovation strategies to improve interoperability of electronic health record communication to improve access, reduce costs, and improve overall quality of care.

Universal access to patient electronic health records (EHRs) in the United States faces significant challenges due to the multiple number of platforms used across the many healthcare systems. The lack of interoperability deters the sharing and utilization of patient information, hindering the full fulfillment of technology’s potential in healthcare [17]. The systematic literature review aimed to uncover what innovative strategies can healthcare leaders implement to improve the interoperability of electronic health record communication between health information systems to enhance data exchange efficiency, accuracy, and security in U.S. healthcare settings. Five themes emerged during the data analysis process that revolved around the research question. The themes included the utilization of blockchain-based EHR systems, the drive of the Cures Act to achieve interoperability, the advent of artificial intelligence and how it can be used in the future, how the Internet of Things drives the industry to strategically enhance the system, and how the value of interoperability drives outcomes. Given the findings of this literature review, there are strategies that healthcare leaders can utilize to improve interoperability within the healthcare system. The awareness of these technologies is crucial for utilizing them to facilitate universal data sharing and achieve high-quality healthcare in the United States.

## 5. Conclusions

This systematic literature review examined innovative strategies to enhance electronic health record (EHR) interoperability in U.S. healthcare by analyzing 24 peer-reviewed studies published between 2020 and 2024. The findings identified five key themes: blockchain-based EHR systems, the 21st Century Cures Act, artificial intelligence, the Internet of Things, and the overall value of interoperability. Blockchain systems demonstrated strong potential to improve data security, integrity, and decentralized access, while the Cures Act continues to shape a regulatory environment that promotes data sharing and reduces information blocking. Artificial intelligence and IoT further support interoperability through advanced analytics, real-time data exchange, and enhanced clinical decision-making.

Despite these advancements, significant challenges remain, including fragmented systems, legacy infrastructure, high implementation costs, and persistent privacy concerns. Addressing these barriers, as we move forward, requires a coordinated approach that aligns technological innovation with policy and organizational readiness. Improving interoperability remains essential for enhancing care coordination, reducing costs, and improving patient outcomes. Continued innovation, policy support, and cross-organizational collaboration will be critical to achieving sustainable interoperability across the U.S. healthcare system.

## Figures and Tables

**Table 1:** Kappa Data.

		Reviewer 2	Reviewer 2	
		Yes	No	Total
**Reviewer 1**	Yes	25	0	25
**Reviewer 1**	No	0	0	0
	Total	25	0	25

One study was later removed due to a retraction from the publisher before publication, which resulted in 24 studies; however, inter-rater agreement displays the original screening set (n = 25).

**Table 2:** Summarized findings of the literature.

Title	Findings
(1) Blockchain-based scalable IoT cloud for electronic health records using horizontal and vertical mining [11]	By integrating with current healthcare systems, blockchain technology and mining techniques are utilized to build an IoT cloud ecosystem that is safe, scalable, and effective for managing electronic health records.
(2) Transforming healthcare data management: A blockchain-based cloud EHR system for enhanced security and interoperability [12]	To guarantee patient data confidentiality, integrity, and availability, this study examines the best blockchain technology, cloud infrastructure, and data management techniques. It also offers insightful analysis and helpful suggestions for healthcare organizations thinking about putting these systems into place.
(3) Blockchain-assisted secured data management framework for health information analysis based on Internet of Medical Things [13]	Decentralized handling of IoMT devices through blockchain integration is a fast-expanding practice. The traceability function of the blockchain ensures that the data used to create the predictive models are valid, resulting in an accurate diagnostic and decision support system in EHRs.
(4) Blockchain-Powered Healthcare Systems: Enhancing scalability and security with hybrid deep learning [14]	This article finds that data integrity, privacy, and interoperability can be guaranteed while doing away with the need for centralized authorities by incorporating blockchain technology into healthcare systems.
(5) Electronic health records and blockchain interoperability requirements: A scoping review [15]	This study indicates that integrating blockchain technology into EHR systems has potential. The acceptance is growing, but there are still a few implementation obstacles to overcome, including those related to standard viewpoints like patient matching issues, security concerns like legal requirements, and architectural perspectives like scalability and performance.
(6) Exploring applications of blockchain in healthcare: Road map and future directions [16]	Interoperability problems, security and privacy concerns, and fragmented data are some of the challenges facing the healthcare sector. By guaranteeing safe, unchangeable storage across several network nodes and enhancing interoperability and patient privacy, blockchain technology presents viable remedies.
(7) Future of blockchain in healthcare: potential to improve the accessibility, security and interoperability of electronic health records [17]	Blockchain technology has the potential to significantly increase data transparency, safety in patient care, healthcare efficiency, and the quality of medical research by enhancing the flow of health information.
(8) Use of semantic interoperability to improve the urgent continuity of care in Danish ERs [18]	The purpose of this pre-study was to create web services using emphasis framing to improve the interoperability of the emergency room’s EHR and the prehospital health record. Emphasis framing and requirements engineering were used in this investigation. To achieve the goal, an iterative linear requirement specification approach was selected as the framework.
(9) Clinical data extraction during public health emergencies: A blockchain technology assessment [19]	Despite the 21st Century Cures Act’s requirements, the readiness assessment for this study revealed advanced analytical competence in hospital institutions and emphasized inconsistent use of the Fast Healthcare Interoperability Resources format across institutions. It is advised to conduct additional testing at more institutions and yearly exercises that make use of the application of data exchange via a blockchain infrastructure to ascertain whether this approach is feasible in the event of a public health emergency and to increase knowledge of the technical specifications for multi-site data extraction.
(10) Blockchain in healthcare and health sciences—A scoping review [20]	The state of research, as this paper illustrates, indicates that a few EHR, PHR, and clinical trial system use cases are now investigating blockchain-based solutions. We discovered few, if any, publications on Knowledge infrastructures, Picture archiving and communications systems, Automated patient diagnostic services, Administrative systems, Population health management systems, and Pharma supply chains, indicating that several other health information system domains are under-explored. In addition to addressing these specific issues, the research agenda must be expanded to include the search for blockchain-based solutions that protect trust by reducing risks both inside and outside the healthcare industry.
(11) Blockchain integration with digital technology and the future of health care ecosystems: Systematic review [21]	The study concluded that blockchains have countless potential advantages, but actual evidence of long-term therapeutic results based on blockchains enhanced and powered by AI and IoT is still lacking. However, putting blockchains into practice as a cutting-edge method of integrating EHRs across the country and handling typical clinical issues algorithmically has the potential to enhance patient outcomes, healthcare experiences, and people’s general health and well-being.
(12) Electronic health record and semantic issues using fast healthcare interoperability resources: Systematic mapping review [22]	Interoperability problems have arisen at two levels because of the growing usage of electronic health records and the Internet of Things (structural and semantic). Standards are necessary for both correctly reading data and properly exchanging it (semantic interoperability). As a result, significant resources have been allocated to enhance the quality of shared clinical data by organizing and mapping it in accordance with the Fast Healthcare Interoperability Resources (FHIR) standard, to promote the semantic interoperability of data exchanged in the healthcare industry.
(13) BlockIoT: Blockchain-based health data integration using IoT devices [23]	The digitalization of patient information has been made possible by Internet of Things (IoT) devices, which have also significantly changed the way healthcare is delivered by enabling things like remote patient monitoring, healthcare decision-making, and medical research. The fragmentation of data across health infrastructures hinders medical data interoperability at the point of care. To bridge this gap, we provide BlockIoT, which leverages blockchain technology to move centralized and previously inaccessible data from medical equipment to EHR systems. This gives providers more information, enabling them to treat patients more effectively.
(14) Proof of concept of scalable integration of internet of things and blockchain in healthcare [24]	To handle potential privacy and security risks for data integrity, the article suggests a novel framework and a special model that combines IoT networks with a blockchain. Data management, device authentication, authorization, and access control are all handled via smart contracts, which play a key role in this integration process. Additionally shared was a new design model for interfaces that integrates both platforms and shows off its improved performance over the previous versions.
(15) Promoting TEFCA with blockchain technology: A decentralized approach to patient-centered healthcare data management [25]	The potential of blockchain technology to advance TEFCA design is explored in this article. Blockchain guarantees data integrity, transparency, and patient privacy by offering an unchangeable and transparent ledger. Given the circumstances, blockchain technology can assist in addressing the difficulties associated with putting TEFCA principles into practice, encourage patient empowerment and control over their health data, better data interoperability, and improve the standard of healthcare.
(16) Beyond compliance with the 21st Century Cures Act rule: A patient controlled electronic health information export application programming interface [26]	A patient’s complete collection of electronic health information (EHI) must be exportable from certified electronic health records (EHRs) according to the 21st Century Cures Act Final Rule. We develop a model to promote regulatory innovation that expands on the Cures Act Rule’s current requirements and moves toward an interoperable, scalable approach. This model facilitates clinical data sharing for better patient care and medical research, encourages the development of an ecosystem of third-party applications, and makes it easier for patients to access their own health information.
(17) Experiences with information blocking in the United States: A national survey of hospitals [27]	The information blocking provisions of the 21st Century Cures Act Final Rule, which outlawed actions likely to obstruct, hinder, or materially discourage the exchange, use, or access of electronic health information (EHI), went into effect for a small subset of data elements in April 2021 and for all EHI in October 2022. The purpose of the poll was to ascertain how frequently hospital administrators believed that certain activities amounted to information blockage. In all, 42% of hospitals reported seeing some activity that they thought was information blocking, and 36% of hospitals that responded said that medical professionals occasionally or frequently engaged in actions that could be considered information blocking.
(18) The 21st Century Cures Act: A competitive apps market and the risk of innovation blocking [28]	A “final rule” and the 21st Century Cures Act both specify standardized procedures for acquiring electronic copies of electronic health record (EHR) data via application programming interfaces. The goal of the regulation is to build an ecosystem of interchangeable, reusable apps that can be developed once and used “without special effort” in any medical system. The final rule has many restrictions pertaining to information blocking, but there is still worry that the commercial practices governing EHR providers and healthcare institutions in the US may impede innovation.
(19) Improving healthcare quality by unifying the American EMR system: Time for change [4]	A collaborative electronic medical record system is a viable way to improve interoperability in the US healthcare industry. The development of a more interconnected and easily accessible patient information network paves the way for a revolution in the provision of healthcare services. To fully realize the benefits of recent improvements in patient care and system efficiency, as well as to sustain the momentum of breakthroughs in healthcare technology, this transformation is essential.
(20) Blockchain and machine learning in EHR security: A systematic review [29].	The study looks at the integration of blockchain (BC) technology and machine learning (ML) to enhance the security and interoperability of Electronic Health Records (EHRs) and highlights challenges in healthcare data management, including privacy, security, and scalability issues. Using a systematic literature review, the authors analyze existing research on BC-based frameworks, federated learning (FL), and deep learning (DL) to improve EHR security. The paper identifies key areas such as secure data storage, interoperability, and privacy-preserving mechanisms through cryptographic techniques and decentralized systems.
(21) Characteristics of a blockchain ecosystem for secure and shareable electronic medical records [30].	The study reviews the potential of blockchain technology to enhance the security, interoperability, and accessibility of electronic medical records (EMRs). Using a grounded theory approach, the study analyzes industry perspectives on blockchain’s suitability for EMR management. It highlights blockchain’s advantages in secure data storage and sharing while addressing challenges such as governance and regulatory compliance. The findings suggest blockchain could revolutionize EMR systems but require further development for widespread adoption in healthcare.
(22) IoT health data in electronic health records (HER): Security and privacy issues in era of 6G [31].	This study investigates the integration of wearable IoT health data into centralized medical systems, highlighting potential benefits for personalized healthcare. With the advent of 6G, increased data exchange rates and connectivity present both opportunities and challenges. It also identifies key security and privacy risks, including data integrity, confidentiality, and regulatory compliance. It also proposes solutions and guidelines for mitigating threats, ensuring secure and efficient handling of patient health data in future healthcare systems.
(23) Survey of interoperability in electronic health records management and proposed blockchain based framework: MyBlockEHR [32].	This study systematically reviews existing literature to explore four key research questions. It evaluates the potential of a blockchain-based Electronic Health Record (EHR) management framework in ensuring privacy protection, access control, and efficient storage. Additionally, it examines the challenges associated with adopting blockchain for EHR management while assessing the current advancements in cross-chain interoperability solutions for securely sharing EHR across different blockchain platforms.
(24) The past, present, and future of the healthcare delivery system through digitalization [33].	The increasing prevalence of chronic diseases and the global spread of infectious illnesses like COVID-19 have highlighted the need for adaptable healthcare solutions. Digitalization has played a crucial role in addressing both longstanding and emerging healthcare challenges. This study examines the evolution of healthcare delivery for cardiovascular and mental diseases, highlighting technological advancements in digital health. By exploring past, present, and future trends, the research presents a roadmap for digital healthcare transformation, emphasizing the importance of secure and accurate health data aggregation to enhance patient care and medical decision-making.

**Table 3:** Frequency of occurrence in the literature.

Theme	Occurrences	Instances of Attributes (n)	Percentage (%)
Theme 1: Blockchain-Based EHR Systems	1, 2, 3, 4, 5, 6, 7, 9, 1, 11, 13, 14, 15, 20, 21, 23	n = 16	67%
Theme 2: Cures Act	9, 16, 17, 18	n = 4	17%
Theme 3: Artificial Intelligence	2, 4, 11, 13, 19, 20, 22, 24	n = 8	33%
Theme 4: Internet of Things	1, 3, 11, 13, 14, 20, 22, 24	n = 8	33%
Theme 5: Value of Interoperability	2, 4, 5, 6, 7, 8, 9, 10, 12, 13, 15, 16, 17, 18, 19, 20, 21, 23, 24	n = 16	79%

## Data Availability

No new datasets were generated. The data supporting the findings of this study are derived from previously published articles cited within the manuscript.
